# More than the Sum of Its Parts: Donor-Sponsored Cash-for-Work Programmes and Social Cohesion in Jordanian Communities Hosting Syrian Refugees

**DOI:** 10.1057/s41287-022-00536-y

**Published:** 2022-05-13

**Authors:** Tina Zintl, Markus Loewe

**Affiliations:** German Development Institute/Deutsches Institut Für Entwicklungspolitik (DIE), Tulpenfeld 6, 53113 Bonn, Germany

**Keywords:** Cash-for-work, Public works, Social cohesion, Social protection, Refugees, Jordan, Middle East and North Africa (MENA), Social transfer schemes, A13, D02, D63, F22, H31, H53, H75, I38, F15, J16

## Abstract

Cash-for-Work (CfW)/public works programmes have gained great interest recently because they can deliver employment and income for vulnerable households, in addition to dearly needed infrastructure. Studying donor-funded CfW programmes for Syrian refugees and their local neighbours in Jordan we show that CfW can also improve social cohesion, which is particularly important in the context of state fragility and migration. The studied programmes strengthen the sense of belonging and horizontal trust of participants and non-participants, refugees and locals, and in particular women. Their effect on vertical trust, however, is more ambiguous because many Syrians and Jordanians attribute positive effects to donor support rather than to Jordanian authorities. We use a mixed method approach including semi-structured interviews with 390 CfW participants, other community members and neutral observers and a quantitative analysis of a survey covering all 1847 participants of one CfW programme.

## Résumé

Les programmes de rémunération du travail ou de travaux publics ont récemment suscité un grand intérêt car ils peuvent fournir des emplois et des revenus aux ménages vulnérables, en plus de permettre la construction des infrastructures urgemment nécessaires. Nous étudions les programmes de rémunération du travail financés par des bailleurs pour les réfugié·e·s syrien·ne·s et pour leurs voisin·e·s en Jordanie, et nous montrons que ces programmes peuvent également améliorer le niveau de cohésion sociale, ce qui est particulièrement important en situation de migration et dans un contexte où l’État est fragile. Les programmes étudiés renforcent le sentiment d'appartenance et de confiance horizontale des participant·e·s et des non-participant·e·s, des réfugié·e·s et des personnes locales, et en particulier des femmes. Leur effet sur la confiance verticale est cependant plus ambigu car de nombreuses personnes Syriennes et Jordaniennes attribuent ces effets positifs au soutien des bailleurs plutôt qu'aux autorités jordaniennes. Nous utilisons une approche à méthodes mixtes comprenant des entretiens semi-directifs avec 390 participant·e·s au programme de rémunération du travail, des membres de la communauté et des observateurs neutres ainsi qu’une analyse quantitative moyennant un sondage auprès de la totalité des 1847 bénéficiaires d'un programme de rémunération du travail.

## Introduction

‘Cash-for-work’ (CfW) or ‘public works’ schemes are a social protection and a labour market instrument. They provide employment and income to the most vulnerable households. In addition, their workers create public goods—usually dearly needed infrastructure such as roads, water pipes, drains, river dams, bridges or school buildings—which also contribute to growth and poverty reduction. This potential double dividend has recently led to a renaissance of CfW in development policy. Famous examples are India’s National Rural Employment Guarantee Scheme and Ethiopia’s Productive Safety Net Programme, which employ 50, respectively, 1 million people on average per year.

Many scholars and practitioners hypothesise (see further below) that CfW programmes also contribute to social cohesion in four ways: first, by reducing poverty, inequality and vulnerability among participants (and possibly also non-participants); second, by creating public goods in their target communities; third, by bringing members of different societal groups together at work; or fourth, by their sheer existence showing that the state authorities care about people. Yet, we lack empirical evidence supporting or rebutting such assumptions.

This article’s aim is to contribute to closing this gap in literature with reference to Jordan. The country suffers from notoriously high under- and unemployment as well as socio-economic insecurity due to widespread informality in employment—something that the Covid-19 pandemic has revealed once more by the fact that few Jordanians lost their employment but many lost their (informal sector) income (Krafft et al. 2021). Even more so, Jordan struggles with the arrival of huge numbers of refugees—recently mainly from civil-war-torn Syria. International donors have implemented numerous CfW programmes since 2016 in Jordan, just like in Turkey, Iraq and Lebanon to provide livelihoods to Syrian refugees and vulnerable Jordanians but also to yield positive effects on the social fabric and economic development of the host communities (BMZ 2018).

So far, little is known on the questions whether CfW programmes in Jordan have effectively contributed to social cohesion, local economic development and gender relations and on how they operate in a refugee-hosting context. This article addresses these questions. In particular, it discusses to what degree and in which way CfW programmes in Jordan have impacted on different attributes of social cohesion in the receiving communities.

Based on a two-year research project^1^ applying a mixed method approach, we find that CfW programmes in Jordan have enhanced key attributes of social cohesion, as defined by Burchi et al. (2022). They strengthened (i) *inclusive identity*, or more concretely a *sense of belonging* to local communities, particularly of Syrian CfW participants and especially women, but also of Jordanian participants and other community members of both nationalities and genders. (ii) *Horizontal trust* in other community members increased, across CfW participants versus non-participants, as well as especially across nationalities and genders. However, (iii) community members’ *vertical trust* in the state and state capabilities developed in a more ambiguous way as people realised that the CfW programmes are exclusively financed, and often run by, international agencies. Our evidence for CfW effects on (iv) *cooperation for the common good* is limited.

In the following, we give an overview over the pertaining theoretical literature (Sect. 2), introduce the country case of Jordan (Sect. 3) and explain the research methodology (Sect. 4) before we present our findings (Sect. 5) and conclude (Sect. 6).

## CfW’s Effects on Social Cohesion: Clues from Literature

This paper is part of a special issue that seeks to fill a knowledge gap about the relationship between social protection schemes—in this case Cash-for Work (CfW) programmes—and social cohesion.

Our understanding of social cohesion follows Burchi et al. (2022), defining social cohesion as “both the vertical and the horizontal relations among members of society and the state as characterised by a set of attitudes and norms that includes trust, an inclusive identity and cooperation for the common good”.

In our analysis, we discern the effects of CfW programmes on the following:

- *horizontal trust*, defined as outgroup trust between different societal groups;
- *vertical trust*, defined as trust between society and the state;
- *sense of belonging* to a community (specified from inclusive identity in the above definition); and
- *cooperation for the common good*, as the preparedness to engage for the society at large.

All these elements have also implications for a given country’s social contract,^2^ which is more effective and robust if social cohesion is strong.

In addition, we look at the effects of CfW programmes on gender roles. Following Cohn (2013, p. 3), we conceive gender as a “a structural power relation […,] a social system which shapes individual identities and lives”. Gender needs to be considered when analysing the different elements of social cohesion. We try to find out whether CfW programmes’ effects on the four elements of social cohesion is as strong, or even stronger, for female participants.

CfW schemes, in turn, can be defined as “public interventions that provide employment to poor households and individuals at relatively low wages” (Gehrke and Hartwig 2018, p. 112). They fall into the category of conditional cash transfer schemes because they provide transfers only to those who are able—but also willing—to do a specific work, which is often physical and done in the public space. This condition serves as a self-targeting mechanism. CfW aims at generating a “double dividend”: to reduce poverty and vulnerability and, simultaneously, create public goods through the work done by participants. Skills training can constitute a third dividend if participants acquire new skills. However, many CfW schemes deliver mainly on one of the dividends: some contribute well to the reduction of poverty and inequality by generating employment for low-income households while the quality of the infrastructure they build is poor because the participants are not sufficiently skilled. Others contribute to poverty and inequality reduction by creating sustainable and useful infrastructure in underserved areas but much less so by providing jobs because the workers are well trained and hence not from the most vulnerable groups of society (Gehrke and Hartwig 2018). Furthermore, CfW is mainly an instrument of passive labour market policies, compensating participants for lack of employment rather than facilitating entry into the regular labour market. Only if CfW mobilises hidden labour market reserves, i.e. activates people who had been outside the labour force, it contributes to active labour market policies.

Some literature also touches on CfW effects on gender roles. De Mattos and Dasgupta (2017) acknowledge that India’s NREGS often constitutes women participants’ first paid job, widens their influence on household decisions and strengthens their sense of belonging perhaps more than the one of men. Yet, they find no sustained effect on later-generations gender roles, as proxied by the oldest daughter’s years of schooling. Hussam et al. (2021) show that female participants in a Bangladeshi Rohingya camp valued CfW programmes because of newly gained financial freedom while male participants benefited more in terms of increased psychological well-being.

While ampler evidence exists on the effects of other kinds of social transfer programmes on social cohesion (Köhler 2021), only few studies look explicitly at CfW’s impact. In post-conflict contexts, CfW raises participants’ opportunity costs of being part of an armed group (Reeg 2017) or diminishes youth disfranchisement and radicalisation (Andrews and Kryeziu 2013), but evidence is so far very limited. Andrews and Kryeziu (2013) outline three ways through which CfW can affect social cohesion: (i) participation in programme design, (ii) temporary participation in the labour market and (iii) strengthened intra-community trust. On the first mechanism they provide positive evidence from Ethiopia and Yemen, yet also warn that community participation may have negative side-effects diverting benefits to local elites (ibid.). Beierl and Dodlova (2022) find that the presence of CfW activities in Malawi correlates positively with the readiness of people to invest time and in kind contributions in public goods and to interact with others from the same or a different societal group. Roxin et al. (2020) find that CfW programmes in Jordan and Turkey have positive effects on the sense of belonging of participants as well as non-participants (non-participants a bit less so in Turkey), nationals and refugees, but also on the horizontal trust between these groups. Babajanian’s study (2012) on the mutual relationship between social protection and social cohesion mentions effects of CfW programmes on social cohesion, yet only in connection to the Indian NREGS’s specific “rights-based framework”.

At the same time, CfW has a potential to contribute to social cohesion also more indirectly as it benefits not only participants but also other people in the community. CfW participants spend most of their income locally, e.g. on food, clothes and housing, thereby increasing the income of other community members who can now spend more on consumption as well (‘multiplier effect’, see Barrientos 2008). Of course, the selection of applicants for participation creates also winners and losers, which may negatively affect social cohesion (Roelen et al. 2022).

Several studies have looked at social protection programmes in refugee settings (e.g. Ovadiya et al. 2015) and most of them provide initial evidence for positive effects on social cohesion. Valli et al.’s (2019) quantitative study on cash and in-kind assistance to Colombian refugees and vulnerable Ecuadorians concludes that refugees’ social cohesion improved while there was no such effect for beneficiaries of the host society. Conversely, a survey by Lehmann and Masterson (2020) finds that UNHCR cash transfers had an effect on the Lebanese host society. These transfers reduced resentments vis-à-vis Syrian refugees because of shared aid and a multiplier effect.

Yet, all these effects are difficult to quantify because they often lack concrete quality indicators and suffer from a split between national migration policies and local practices (Ozcurumez and Hoxha 2020).

## Social Cohesion and Social Protection in Jordan

Jordan’s history since independence in 1946 has been marked by several waves of migrant and refugee arrivals from neighbouring countries. In particular, large numbers of Palestinians came to Jordan in 1948/1949 and 1967. Though only part of them and their descendants hold Jordanian citizenship, they now account for more than half of Jordan’s citizens. On top of this, there are refugees from the Lebanese civil war (1975–1990) and the US intervention in Iraq (after 2001) as well as migrant workers, in particular from Egypt. After the so-called Arab Spring 2011, further refugees came from Libya, Yemen, Sudan and, in largest numbers (probably 0.6–0.7 million^3^), Syria.

### Syrian Refugees and Their Effects on Social Cohesion in Jordan

Only 20% of the Syrian refugees live in designated refugee camps, the vast majority lives in Jordanian cities, towns and villages (World Bank 2019). There they add to existing socio-economic hardship and strained public infrastructure in Jordan in terms of water and sewage, housing, unemployment and underemployment, health and education.

The presence of the refugees has continuously put social cohesion within Jordanian communities and Jordan’s national identity to a test. An online survey among Jordanian and non-Jordanian youth showed that horizontal trust within personal networks slightly decreased between 2015 and 2017 (Kuhnt et al. 2017). At the time of our research, social tensions affected many schools though, or because, many Syrian refugee children are schooled in separate afternoon shifts. These tensions resulted in feelings of isolation among refugee children and youth, difficulties in access to education and school drop outs (Mercy Corps 2012; Grawert 2019). In 2017, 20% of Syrian children at primary school age (5–12 years) and 82% of those at age 16–17 were not enrolled in schools, while the respective rates of Jordanians were just 2, respectively, 21% (MOE and UNICEF 2020).

Reportedly, external aid has in some cases contributed to the weakening of social cohesion. This is especially the case when the distribution was perceived as unfair and tainted by corruption—an impression received by more women than men and by more Jordanians than Syrians (REACH and British Embassy 2014). Rumours circle that refugees make money by re-selling tents, blankets or cooking utensils donated by foreign donors. Competition for affordable housing intensified and rental prices increased because of housing subsidies paid to Syrian refugees (Grawert 2019, p. 23). In some localities, for instance Al-Mafraq (Grawert 2019; Mercy Corps 2012), open clashes broke out (UNHCR et al. 2014).

Still, social cohesion remains surprisingly sturdy in most local communities. Tribal relations, common language, cultural ties and geographic proximity but also age and similar interests seem to be more influential drivers of mutual trust than nationality or religion (Kuhnt et al. 2017). Tribal relationships extend across borders and solidarity for the newly-arrived was generally high, even by former Palestinian refugees (Grawert 2019). A survey by Alrababa’h et al. (2021) found that cultural ties and humanitarian concerns are more decisive for Jordanian hosts’ attitudes towards refugees than economic hardship or strained infrastructure. However, due to deteriorating living conditions and more frequent struggles over the distribution of resources, there is a veritable risk that social cohesion becomes increasingly instable.

The social fabric of host communities has also been influenced by the fact that 22% of the Syrian households in Jordan are female-headed (Tiltnes et al. 2019). Many women sought shelter alone or only with accompanying children as widows or as their husbands stayed back in Syria (UNHCR et al. 2014). Female refugees are particularly vulnerable as they are marginalised twofold, as women and as refugees. One survey (GBVIMS 2017) estimates that a third of Syrian refugee women experienced sexual violence and more than half experienced emotional abuse—and these figures might even underreport because of the strong stigma surrounding the topic (UNHCR et al. 2014). Refugee girls are often married off at a young age *inter alia* to secure their own and their families’ livelihoods (Tiltnes et al. 2019; UNHCR et al. 2014).

### Countering Weak Social Protection: CfW and Other Social Transfer Programmes

At first glance, Jordan’s public social protection system is rather advanced. It includes contributory social insurance and non-contributory social transfer schemes.^4^ At the time of our research,^5^ however, the latter covered only Jordanians and long-term residents with the exception of a basic health care fee waiver extended in 2019 to cover low-income registered Syrian refugees as well (Hagen-Zanker et al. 2018). Therefore, the Government of Jordan called on the international donor community to provide social protection for Syrian refugees, as had been, and continues to be, the case with UNRWA’s support for Palestinian refugees.

With the Jordan Compact of 2016, the Government of Jordan and the international community agreed that *inter alia* Jordan will promote access to the formal labour market for Syrian refugees in specific economic sectors such as food processing, handicrafts and tailoring, whereas international donors will provide further social protection for Syrian refugees.

As a consequence, international donors set up social transfer programmes for Syrians in Jordan. These are, just like the UNRWA-support for Palestinians, completely donor-financed. Typically, they are implemented by international or national non-governmental organisations or public institutions with rather lose coordination with national ministries. Foreign donors (mainly UNHCR and UNICEF, but also the World Food Programme and several NGOs) run unconditional cash transfers,^6^ voucher, winterisation, education, vocational training, employment and empowerment schemes for Syrian refugees. In line with the calls for strengthening host communities’ resilience, as formulated by the Regional Refugee Resilience Plan or the Jordan Response Plan (REACH and British Embassy 2014), most of these schemes include a quota for vulnerable Jordanians as the Government of Jordan stipulated a 30–50% minimum share of Jordanian recipients (Röth et al. 2017).

Since 2016, CfW programmes account for the largest share of the funding of these programmes. Between 2016 and 2019, their budget reached about 300 million EUR (Loewe et al. 2020, Table 1). Many projects are run by WFP, UNHCR, UNICEF and other multilateral and some bilateral donors. Yet, the bulk is financed by Germany through the so-called “Partnership for Prospects” initiative, which explicitly sets three goals: easing Syrian refugees’ and vulnerable Jordanians’ financial stress, strengthening social cohesion by reducing competition in the labour market and promoting female labour force integration (BMZ 2018).

**Table 1:** Research sites and CfW programmes by donor and main activity covered by qualitative survey

Region	Governorate	Locality	Number of interviews	CfW programmes	
				Funding and implementing agencies	Main activity
North	Irbid	Kafr Ṣawm	55	GIZ with World Vision	Water dam rehabilitation
		Kafr Asad	59	World Food Programme with An-Nağmah	School rehabilitation
				GIZ with Norwegian refugee council	Waste collection
	Al-Mafraq	Al-Mafraq	18	KfW/ILO	Waste collection
		Irbid Highway	10	KfW/ILO with local contractors	Waste collection
		Umm al-Jimāl	44	GIZ with Oxfam	Waste collection
	Az-Zarqā’	Al-Azraq	35	International Cooperation and Development Fund (Taiwan) with Action against Hunger	Waste collection
				GIZ	Nature reserve rehabilitation
Centre	Al-Balqā’	Tal ar-Rummān	6	GIZ	Water dam rehabilitation
		Dayr ‘Allā	24	GIZ with National Agricultural Research Center	Agricultural work
				GIZ	Waste collection
South	Al-Karak	Faqū’a	30	Norway/ILO with Agricultural Directorate	Tree planting
				GIZ with Danish Refugee Council	Water dam rehabilitation

In terms of geographical scope and main activities, CfW programmes in Jordan have a broad range. They are carried out in camps and host communities, and coverage is high—a fifth of all Syrian refugees in Jordan had participated in a CfW programme at least once within the year preceding the study by Tiltnes et al. (2019). Most CfW sites outside camps are located in Jordan’s northern governorates (83%) as most Syrian refugees settled near the Syrian border, but some activities are also in the central (10%) and southern (7%) governorates (Loewe et al. 2020, Fig. 7). CfW activities in Jordan include the building and maintenance of ‘grey’ and ‘green’ infrastructure (streets, dams, schools, health clinics; water reservoirs, irrigation systems, municipal parks and ecosystems), waste management and the intensification of agriculture.^7^

All CfW programmes employ women and men. Sometimes, women do the same kind of work as men, or similar work excluding hard physical labour. Sometimes, however, they have very different tasks such as cooking meals for the male participants, who do the physically more demanding jobs. Contract durations typically range between 2 weeks and three months at 20 working days, while they are six months at 16 working days in other projects. To harmonise CfW operations, for instance in regard to the level of salaries, a donors’ CfW coordination group is in place.

## Research Methodology

Following the existing literature (Sect. 2), we hypothesise that CfW programmes in Jordan have a positive impact on all four components of social cohesion.

Our two-year research project applies a mixed method approach. It is predominantly based on (i) semi-structured interviews with 281 CfW participants and non-participants at nine CfW sites all over Jordan and (ii) qualitative interviews with 99 neutral observers^8^ at the local and national levels. Further insights are drawn from (iii) four group discussions,^9^ all conducted in Amman in 2019 and (iv) the quantitative analysis of a census of all 1847 participants of one specific CfW programme in 2019 and 2020—what we will call the GIZ Post-Employment Survey (GIZ 2019, 2020) in the remainder of this chapter).^10^ The bulk of the research (i-iii) has been conducted by a team of six researchers from the German Development Institute/Deutsches Institut für Entwicklungspolitik (DIE) during a 3-month field stay in early 2019 (Loewe et al. 2020).

We decided for a predominantly qualitative approach supplemented by a quantitative survey because social cohesion is a complex phenomenon consisting of several immaterial elements, which are hard to measure as they rely on people’s perceptions. We focussed on communities, not camps, with relatively large CfW projects (some even with two or three projects) in fairly isolated communities in order to capture community effects as good as possible and be able to attribute them to the respective local CfW project.

We selected suitable sites (Table 1) based on, first, a mapping of all 402 current CfW projects provided by local implementing agencies with all of their characteristics and, second, the geographical spread of CfW projects and Syrian refugees in Jordan. We controlled against a possible selection bias by using different points of access to our interviewees, through (i) local CfW implementers, (ii) community-based organisations and (iii) randomly approaching community members in public places. While we aimed to interview even numbers of Jordanians and Syrians, participants and non-participants, men and women, this was not always possible.^11^ For example, some assumed non-participants turned out to be participants of earlier CfW programmes; and some groups—for instance, female non-participants—were more difficult to access. Furthermore, we took care to include CfW programmes with different activities, by different funding agencies and with different design features. It should be noted though that the existing CfW programmes are sufficiently similar to allow for comparison even though there is a bit of variety in several design features. We are aware that by relying mainly on qualitative interviews we gathered only a medium-sized sample, yet are convinced that, by doing so, we gained better insights in refugees’ and hosts’ situation and in (perceived) changes in social cohesion.

## Findings: Jordan’s CfW Programmes and Social Cohesion

“We don’t think of ourselves as Syrians or Jordanians here” (non-participant, Kafr Ṣawm).

In this section, we assess the CfW programmes’ impact on different elements of social cohesion in Jordanian host communities. In addition to the here discussed effects, CfW programmes positively influence local economic development and thus indirectly improve social cohesion. Just like other cash transfers, CfW income is spent and re-spent within local communities and spawns a multiplier effect (Loewe and Zintl 2021). Such cash injections strengthen horizontal trust between community members.^12^ Furthermore, CfW participants gain new skills and work experience but the effect on employment rates is less noticeable than the effect on social cohesion, because Jordan’s labour market is very tight and access to it restricted (ibid.).

Since we first asked all interviewees at community level to describe social cohesion *irrespective* of CfW programmes,^13^ we start by the observation that our interlocutors by and large confirmed the current state of Jordanian–Syrian social cohesion, as described in Sect. 3. A majority saw social cohesion between community members of both nationalities as good and emphasised that Jordanians and Syrians share similar social norms and traditions, a common history and—especially along the Jordanian–Syrian border—common tribal relations and intermarriage. Two Jordanians remarked: “There is no discrimination” (shopkeeper, Faqū’a); “we are one and we all face the same challenges” (non-participant, Kafr Ṣawm). A Syrian man told us “We’re very connected people. […] We are one over here” (non-participant, Al-Azraq). None of our interviewees told us about substantial tensions in our local research sites. Yet, some Syrian women described violent or discriminatory incidences at their kids’ school. “My children sometimes get abused because we are Syrians. […] My older son has got a broken arm because he got into a fight” (non-participant, Kafr Ṣawm).

Most interviewees expressed a strong *sense of belonging* to the community, though many Syrians continue to miss their native country: “Yes, I belong here. But there is nothing like home” (non-participant, Ḥawfā); “People are very welcoming. […] But our heart is in Syria” (non-participant, Al-Azraq). Only a small minority of interviewees said they felt they do not belong to the community.

Almost two-thirds of the respondents in our qualitative interviews being asked about *horizontal trust* described relations between both groups as good. The share was higher among Jordanians and Syrian women than among Syrian men. The remaining third of respondents described horizontal trust as mediocre or poor.^14^ Another sign for intact horizontal trust is the widespread practice of local shopkeepers to grant informal loans to both Jordanian and Syrian customers. “Loans play a big role. […] All people take loans” (shopkeeper, Deyr ‘Allā). These vastly positive findings are not trivial because social cohesion between refugees and locals is often weak, as Roxin et al. (2020) show for Turkey.

However, interviewees were very concerned about insufficient infrastructure.^15^ Syrians mainly complained about accusations made by Jordanians: “They blame us, the Syrians, […] for the increases in the rents, the electricity prices, anything” (non-participant, Faqū’a). And Jordanians expressed these very accusations: “Everyone lives their life here, but they [the refugees] took job opportunities from Jordanians” (non-participant, Tal al-Rummān). Several Jordanian interviewees maintained that Syrians have an undue advantage because of the international aid they receive.

Nevertheless, most interviewees emphasised that, on a personal level, they get along well with individuals of the respective other nationality. “We can separate between work and life. Work is one thing, but our relations in general are very good” (shopkeeper, Faqū’a). Some interviews pointed to the fact that negative feelings about the respective other group are more likely to prevail if both groups have no or little contact. CfW programmes encourage contacts between the groups and thereby improve different elements of social cohesion.

### CfW and Sense of Belonging to the Community

“Yes, the programmes improved the relations. […] The Syrians […] became part of society” (non-participant, Kafr Ṣawm).

According to our findings, CfW programmes in Jordan strengthen community members’ sense of belonging. 80 respondents explicitly talked about whether or not CfW programmes made them feel integrated in their local community: 30 interviewees stated that the programmes had helped them to feel more attached to their community—a few (5) had previously felt not well-integrated, for most (25) the programmes rather reinforced a general feeling of belonging. Participants of both nationalities mentioned that their sense of belonging had improved without having been prompted, so the evidence is quite strong. 50 respondents declared that the programmes had not had any tangible effect on their feeling of belonging—for a majority (41) it had always been good, for others (9) it was still bad. No respondent mentioned any negative effect of CfW programmes on their sense of belonging. Non-participants of either nationality reported less often an improvement. These findings support two assumptions: (i) if, like in Jordan, the sense of belonging of locals and immigrants is already quite strong only the experience of working with people from the respective other group can still make a tangible difference^16^; (ii) having a job helps to feel integrated (Wietzke 2014). Several interviewees strengthened the second assumption by pointing out that the CfW work as such—indeed having any kind of work—makes people feel part of their respective local community. A Syrian non-participant stressed: “At the beginning, I felt as an outsider. But now, I feel part of society. This is mainly because I’ve got a job. It helped me a lot” (non-participant, Kafr Asad).

CfW programmes fostered an inclusive identity especially by providing a joint work experience. By comparison, knowing that such CfW programmes exist or the created infrastructure had no such effect. The latter is rather surprising, as not even the creation of municipal parks or upgraded school buildings to be jointly used by the public, had a discernible effect on whether or not Jordanians or Syrians in our sample felt they belonged to the community. In Quweirah, a quite dreary desert settlement on a highway in Southern Jordan, a CfW project created a restful and shadowy municipal garden, so people can meet and enjoy this one beautiful place in town together. Employment, in contrast, gives weakly integrated individuals a meaningful activity. CfW design choices like duration of contracts or possibilities to re-apply to another CfW job play a role. Long or repeat contracts enable participants to take decisions on life events, which makes them feel integrated in society: “Through the project[’s income] I have been able to get married” (male participant, Umm al-Jimāl).

The inclusive effect of having a paid job was particularly important for women. Through their CfW participation, women interact with the wider community beyond traditional gender roles, which foresee household chores and childcare. “[The CfW programmes] have changed the attitudes that the woman is an active part of the society […] The project has transformed the whole community” (male participant, Umm al-Jimāl). Several interviewees remarked that the CfW programmes increased the general recognition of females as part of society: they employ a relatively high share of women, often let them do similar work to men, and thereby demonstrate that women can make more or less the same contributions as men. “They made us feel the importance of the women’s role in community. […] In the past there were plenty of taboos about women going out and working. But now this has changed” (participant, Umm al-Jimāl). Participating in CfW programmes and in associated skills trainings strengthened women’s self-confidence and thereby their standing within the community. “It helped to shape my personality; I became more confident. I can now be an active member of the society” (female participant, Kafr Asad).

While a paid job can improve women’s feeling of belonging, it is extremely difficult for them to find employment; CfW programmes are a realistic point of entry into the labour market for women. Especially in rural Jordan there are very few job vacancies but numerous unemployed men, who are more flexible and mobile^17^ in their job search than women: “There are very few jobs in town; and often, these few jobs are reserved for men” (female participant, Al-Azraq). CfW programmes, in contrast, need to fulfil gender quotas so success rates for women applicants are high. CfW participation often constitute women’s first formal employment ever.

Cultural norms play a decisive role whether women take up paid employment and by doing so feel more integrated into their community. In Jordan, CfW participation confronts reservations held by many Jordanians and Syrians against women in paid employment.^18^ The majority of the interviewed women and men considered female labour force participation as acceptable, at least under specific conditions, like ensuring that work tasks and work locations are suitable for women (see below). However, other interviewees of both genders told us that reservations about female labour participation are so strong that many families consider this option only during financial hardship. Intra-household relationships, while extremely important for female empowerment, evolve slowly and are difficult to change. Women may stop working once the economic situation improves. One woman told us: “I do not like it because it is forced by the economic situation. I would prefer to stay at home and care for my children” (female non-participant, Kafr Asad); her sense of belonging to the local community might actually be higher if she had the traditional role as housewife. CfW participation thus provides an additional, but not irrevocable avenue to changed gender roles and women’s sense of belonging.

### CfW and the Readiness to Cooperate for the Common Good

“[Because of the CfW programme] others became aware of the environment. They started using the trashcans” ( participant, Kafr Asad)

Because of refugees’ dire economic and income situation, we did not expect to find strong evidence for CfW strengthening the readiness of people to engage for the common good. Beierl and Dodlova (2022) detail such evidence for a CfW programme in Malawi.

We found an effect only in regard to more environmentally conscious behaviour. CfW programmes in the waste sector had a positive effect on participants’ and community members’ environmental awareness and triggered a higher motivation for proper waste collection. CfW participants led by example and affected the behaviour of community members who appreciated the cleaner streets and recognised the necessity of recycling waste. Especially children and youth were guided to contribute in this way to the common good: “Children in school observe people cleaning and separating waste and thus also act on it and realise that putting waste in the environment is bad” (local expert, Umm al-Jimāl).

Whether or not CfW programmes motivate participants to contribute to, and advocate for the common good depends considerably on the amount of time or in-kind resources such engagement requires—binning your waste does not consume additional time or resources, but organising community meetings does. Participatory project designs, longer CfW contracts or agreements with local communities to service the new infrastructure create a degree of ownership among participants or the community at large^19^ and, thus, may raise their engagement for the common good.

### CfW and Horizontal Trust Between Community Members

“I worked with Syrians and we built friendships” (participant, Deyr ‘Allā).

Participating in CfW programmes does not only trigger a sense of belonging, as was detailed above, but also deepens horizontal trust. In Jordan, not only the joint work in mixed Syrian–Jordanian teams, but also the fact that refugees work to create or maintain public infrastructure boosts trust within communities.

Working with individuals from the respective other nationality gave the largest boost for “outgroup” horizontal trust between Jordanians and Syrians. Almost half of the interviewed CfW participants stated that CfW programmes had further strengthened horizontal trust. A fifth told us that the programmes had *not* improved horizontal trust but all except two individuals considered Syrian–Jordanian mutual relations so strong they could not be strengthened further. Most interviewees made their statements spontaneously, without being prompted, thus showing that the programmes’ effect on horizontal trust is considerable.

As reasons for this positive effect, participants pointed to work-related interaction (exchange about skills and working techniques; team work to get work tasks done) and to the time spent together (shared meals; conversations about co-workers’ interests and values; leisure activities after work). Syrians and Jordanians learnt about each other’s needs and hardships: “There should be more work that supports both Jordanians and Syrians, we should work with them together so we can better understand their situation. Sometimes you feel that Jordanians are like us, they don’t have income either” (participant, Umm al-Jimāl).

The vast majority of interviewees felt that working in mixed-nationality teams had positive effects.^20^ “I love that I work in mixed groups of Syrians and Jordanians. […] Many other workers were sceptical in the beginning about cooperating with Syrians. But the project changed their mind” (Jordanian participant, Kafr Asad). To best facilitate social cohesion, CfW programmes should assign mixed-nationality work teams and foster reciprocal learning.

Many Jordanians and Syrians built friendships during their joint CfW participation, which often continued thereafter: “We are like brothers. Even when the programme finished” (participant, Deyr ‘Allā). In the GIZ Post-employment Survey (GIZ 2019) 86% of all respondents stated they had made friends with people of the other nationality. More Syrians than Jordanians (94 versus 78%) and more women than men (94 versus 83%) felt this way. Likewise, in a survey by NAMA and ILO (2019), 91% of respondents affirmed they had built a friendship with co-workers, including people of the respective other nationality, and 83% felt that their CfW participation had helped to reduce tensions between Jordanians and Syrians.

For non-participant community members, the positive effect of CfW programmes trust was smaller but still notable. Many but not all of those non-participants who found that the CfW programmes had improved horizontal trust in their community drew on positive experiences they had heard from participating relatives, friends and neighbours. A Jordanian community member commended: “The programmes had a lot of impact on the social relationships between Syrians and Jordanians. [… They] got better due to the programme. […] The truth was revealed that the Syrians can also work for the community” (non-participant, Kofr Asad). The generally positive effect on non-participants’ trust was confirmed by another study (Roxin et al. 2020).^21^

Interviewees, both CfW participants and non-participating community members, lauded the fact that both Syrians and Jordanians were eligible for the CfW schemes. Not a single interviewee claimed the scheme should have employed only people of their own nationality. Still, a handful of Jordanian interviewees found it unfair that Syrians, representing less than half of their community’s population, got half of the CfW work contracts. In fact, this envy proves that participating in CfW programmes was seen as a great opportunity and did not stigmatise vulnerable people relying on the programmes, as occasionally reported for other social protection schemes (Burchi et al. 2022). The recruitment of both nationalities also calmed accusations about refugees taking away opportunities and scarce resources. A Jordanian women pondered: “The Syrians have taken the jobs that should belong to Jordanians [… But] we have to accept that they are here. And we have to accept that the CfW programmes employ Syrians as well because without the Syrians, we would never have got the programmes here in Jordan” (participant, Deyr ‘Allā).

Jordanian and Syrian interviewees complained about unfair recruitment practices because of favouritism, but saw no negative repercussions on horizontal trust. *Wasţa* [Arabic: connections] played a role in the selection of both Jordanians and Syrians (e.g. Syrian participant, Kafr Asad), but reportedly was more of a problem in regard to Jordanian CfW participants—as the vast majority of Syrian refugees is vulnerable anyhow: “The Syrians […] were selected in a fair way but for the Jordanians it was mainly by *wasţa*” (Syrian participant, Kafr Ṣawm; similarly, Jordanian participant, Faqū’a). This preferential treatment of some Jordanians, however, seemed not to affect community members’ horizontal trust in the respective other nationality but rather vertical trust, as will be detailed below.

CfW programmes also increased the horizontal trust of women—yet, in a somewhat different way. Both mixed-gender or gender-segregated teams fostered more trusting relationship. Some female participants found that working in a mixed-gender team had been a very good experience while others, especially in more rural and conservative areas, preferred all-female teams.

Female interviewees valued the interaction with others at the workplace. CfW participation allowed them to talk with other community members with whom they have no or little contact in everyday life. “The programmes have many benefits. They are entertaining. And they are bringing people together to do more productive things” (female non-participant, Kafr Ṣawm).

In regard to gender, trust is often linked to behaving in a way that respects cultural norms. For our interviewees the question whether the type and location of work was suitable for women was much more important than the gender composition of work teams. Men and women agreed that men should be assigned the physically demanding work, such as carrying stones or handling heavy machinery. Women were also sceptical about work tasks that were not physically demanding but traditionally carried out by men, such as light construction work (female participant, Kafr Asad). Similarly, most interviewees thought that working in waste collection was not acceptable or “embarrassing” (participant, Irbid highway) for women. CfW activities raising awareness about waste recycling were considered more suitable for women, because it is culturally acceptable for women, but not for men, to visit private households and speak to housewives, who are at home alone and usually responsible for dealing with household waste.

Generally, and in line with other studies (e.g. World Bank 2018), work tasks to be accomplished indoors were considered more suitable for women than those to be done in the public space (e.g. participant, Irbid highway). Especially male but also female interviewees found it inappropriate for women to be in contact with strangers in workplaces. Women recounted that “to go out and work” (Umm al-Jimāl) was their main challenge against CfW participation. Yet, CfW contributes to changing stereotypes of which work is socially acceptable for women.

Moreover, because donor-run CfW programmes respect labour rights and guaranteed working hours, many interviewees considered them a safer work environment than other available, often informal work. “I tried working outside, but it did not work out. Here [in the CfW programme] it is better. Over here, the treatment is much better. We are taken care of” (female participant, Deyr ‘Allā).

The Post-employment survey (GIZ 2019) illustrates the same point. When asked about future plans for after their CfW participation, women more often than men stated that they would look for another CfW opportunity (73 vs. 71%) rather than for a job in the formal (26 vs. 34%) or informal (3 vs. 5%) sector or further training (10 vs. 15%). This preference was most pronounced among Syrian respondents.

### CfW Programmes and Vertical Trust in Authorities

“We trust you [foreign donors] more than we trust the government” (local expert, Kafr Asad).

Our results show that, if implemented well, CfW programmes can foster participants’ and even non-participants’ vertical trust. However, there are two important qualifications: First, what does ‘implemented well’ mean (i.e. which features are particularly important for CfW programmes to foster vertical trust)? Second, vertical trust *in whom* can they foster?

Whether and to what extent CfW generates vertical trust depends on programme design choices, mainly whether targeting is perceived as fair and to what extent programmes are run in a transparent manner. Respondents of the survey criticised CfW mainly for the quality of meals provided (22%), the level of wages (16%), transportation (11%) and working hours (9%) but less so for issues of bad implementation (in terms of feed-back mechanisms: 8%, the behaviour of the supervisor: 5%, the behaviour of the employer: 3%). The selection of participants and the possibilities of participation in project design could not easily be ticked as a source of dissatisfaction but only one person complained about the selection process in the open section on recommendations for improvement (GIZ 2019; GIZ 2020).

Interviewees who saw the selection of participants as fair expressed higher levels of vertical trust in authorities responsible for the CfW programmes than those who perceived targeting as unfair and intransparent. Fair recruitment of CfW participants requires that all eligible persons know about the work opportunity and that participants are selected based on transparent and fair criteria. The interviewees stated that finding out about CfW job opportunities had been least problematic. The information about the programmes and how to apply had been widely available through online and print advertisements or word-of-mouth communication (e.g. participant, Dayr ‘Allā).

In contrast, several interviewees criticised the selection criteria and recruitment of CfW workers as intransparent and subject to favouritism. “The […] project is running through *wasţa*; I know people that have been working there for years. […They] have *wasţa* to be able to do [more than one CfW contract]” (participant, Kafr Asad). Most CfW programmes started to form local selection committees at each field site, deciding recruitment based on an elaborate list of criteria of vulnerability and, sometimes, qualification. For instance, in two sites, representatives of the municipality as well as vulnerable community members or representatives of local charitable organisations were part of such committees (local expert, Al-Azraq; local expert, Kafr Asad).

Local participation in project design can build vertical trust, too. Some CfW programmes we investigated facilitated discussion about possible infrastructure to be created, but this is also subject to planned CfW activities—community parks lend themselves easily to community participation while highway maintenance hardly does. Throughout, interviewees valued this opportunity and expressed higher vertical trust in planning and implementing agencies. In reverse, CfW programmes with activities designed in a top-down manner were criticised: “We wish to have more say in where the projects take place. It was not clear to me what power we have. […] We did not know whom to contact to ask for changes” (local expert, Faqū’a).

Problematically, CfW in Jordan sometimes fosters vertical trust not in local or national authorities but in foreign donors, who fund the programmes and either implement or oversee the implementation of them. Often, the only active role of national Jordanian authorities is to grant work permits. On local level, municipalities delegate representatives to participate in local selection committees. Yet, exactly the role played by these representatives was frequently criticised because of *wasţa*, rather damaging than building vertical trust. In Kafr Asad, for instance, a local expert recounted:

“The selection process was very unfair. I’m saying that it is not the fault of [the implementing agency]. I register […] with the *baladiyya* [Arabic: municipality], to be employed by a CfW project […]. If I work in the *baladiyya*, I can tell my friends to apply for the programme” (local expert, Kafr Asad).

One interviewee complained that “outsiders should manage the programmes. It is not good when locals manage it. The money is lost due to corruption” (participant, Faqū’a). Other interviewee expressed their relief that foreign donors were overseeing the CfW recruitment process because “if organisations ask people to apply through the municipality, *wasţa* will become a problem. Then, from the same household there are many people working, but sometimes in other households, no one would find work” (non-participant, Umm al-Jimāl).

## Discussion and Policy Recommendations

The case of Jordan shows that CfW programmes can be an effective instrument of support for both, vulnerable individuals and (host) communities. They help to reduce poverty, inequality and vulnerability by providing paid employment and infrastructure. Households in remote and underserved areas gain access to key social services, markets, information and institutions. But they contribute also to social cohesion in all its dimensions and thereby to political stability (Burchi et al. 2022). By integrating refugees into society, reconciling different societal groups and empowering women, CfW strengthens the sense of belonging and horizontal trust of participants and non-participants, nationals and refugees. To some degree, CfW programmes also foster community members’ readiness to cooperate for the common good.

Of course, the size of these effects depends on the local context and program design.

In Jordan, CfW programmes benefit from the fact that participating Jordanians and Syrian refugees share joint cultural values and a common language. Rather favourable initial levels of social cohesion already help to counteract socio-economic instability. The successful implementation of CfW in Jordan indicates a reverse causality: high levels of social cohesion contribute also to the effectiveness of social protection (Burchi et al. 2022).

**Fig. 1:**
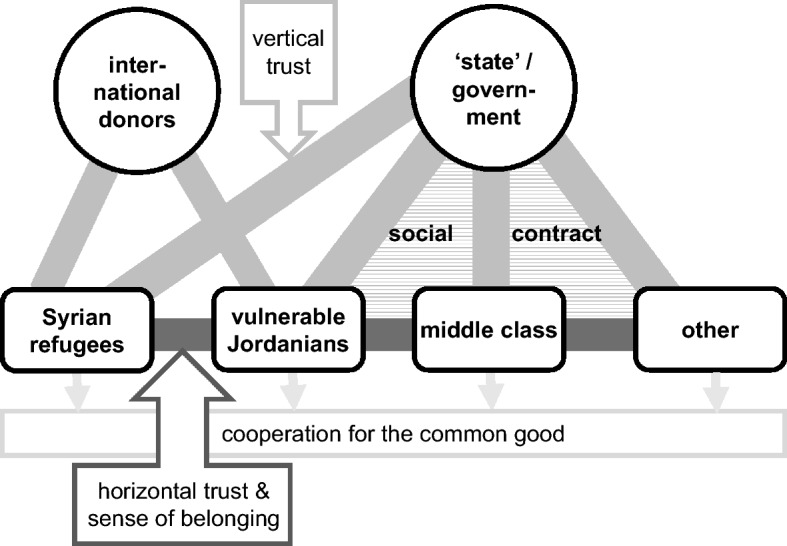
Elements of social cohesion and the risk of diverting vertical trust to donors. Source: authors

Vertical trust also improves because of CfW programmes in Jordan, yet not necessarily trust in local authorities. The large number of refugees on Jordanian soil necessitates an international shouldering of costs. In addition, Jordan’s authorities prefer not to implement large-scale social protection schemes for Syrian refugees because doing so could jeopardise social cohesion inside the Jordanian population due to jealousy or xenophobia (Alrababa’h et al. 2021). The fact that donors have so far funded and run the CfW programmes in Jordan allows the country’s government to disassociate from all of their negative effects. However, the government can also not claim tribute for the merits of these programmes, which ‘diverts’ CfW’s positive impact on vertical trust of at least parts of society (perhaps mainly the Syrian migrants) away from the Jordanian state towards foreign donors. This has significant implications for the social contract in Jordan (Fig. 1), as foreign donors ‘substitute’ for the government by fulfilling an important part of its obligations to provide social protection.

If the government of Jordan decided to implement at least part of the CfW programmes itself, it could rightfully assume responsibility for all inhabitants. It could gain legitimacy by demonstrating that it is capable of operating CfW programmes and implementing social policies in a coherent and efficient manner. To do so, it could build on the existing donor-run programmes—just as it “piggy-backed on [donors’] administrative mechanisms” in its social protection answer to the Covid-19 pandemic (Hagen-Zanker and Both 2021)—as well as on international experiences such as the Indian NREGS.

However, Jordanian authorities’ low initial levels of vertical trust due to allegations of corruption and favouritism render such a strategy challenging and risky. The responsible implementer gains or loses the population’s vertical trust depending on whether or not CfW programmes are run successfully.

Overall, CfW programmes are clearly recommendable instruments also in contexts of crisis and migration and implementers should build on experiences made with the design of CfW in other countries. Best practices that strengthen social cohesion are, for instance, participatory planning of activities, mixed-nationality teams, quotas for women participation or skills trainings that foster reciprocal learning. CfW programmes can clearly deliver several things at once even though there is a certain trade-off between their direct effects: In addition to the “double dividend”—(i) the generation of income for unskilled workers in order to strengthen social protection and (ii) the creation of good-quality infrastructure that actually requires a minimum of skilled labour—there are indirect, immaterial effects on communities’ social cohesion. These immaterial effects are likely more permanent, though more difficult to identify, than the material effects as they leave a more lasting impression on people’s minds. Social cohesion, yet again, facilitates economic activities and the successful implementation of social protection schemes. In that sense, social protection and social cohesion are interdependent. Cash-for-Work programmes that start such a virtuous circle are truly more than the sum of their parts.

## Footnotes

1 We are indebted to our research team members Jörn Fritzenkötter, Verena Gantner, Regina Kaltenbach and Lena Pohl. Loewe et al. (2020) provides a full report of our research project, with an additional focus on CfW programmes’ effects on communities’ local economic development.

2 A social contract includes the “entirety of explicit or implicit agreements between all relevant societal groups and the sovereign (i.e. the government or any other actor in power), defining their rights and obligations towards each other” (Loewe, Zintl and Houdret 2021), so mutual trust affects whether it is considered functional by all contracting parties.

3 See UNHCR (2020). Other, perhaps politically motivated, sources estimate the number of Syrians in Jordan at 1.2–1.4 million but this figure is inconsistent with demographic and labour market surveys, see Krafft et al. (2019).

4 Jordan spends a large share of its GDP (12 per cent in 2012, Loewe and Jawad 2018, p.10) on social protection and health, but mostly on public sector pensions and health care, thus excluding the poorest parts of society that work in the informal sector. Less than 1 per cent of GDP goes into social transfer programmes (Loewe 2019).

5 These schemes were only extended in 2020, due to pandemic lockdowns (Hagen-Zanker and Both 2021).

6 By 2020, these payments constitute the only household income for over half of the Syrian refugees (Jones 2021).

7 One project providing teacher salaries also operates under the label Cash-for-Work, though it does not fulfil the usual criteria of CfW programmes because (i) it provides skilled work and generates higher wages and (ii) it does not create infrastructure. Furthermore, since this occupational field is not open to non-Jordanians, all its direct beneficiaries are Jordanians. (For more information see Roxin et al. 2020).

8 Neutral observers on the local level were, for instance, mayors, tribal leaders, NGO workers or school directors. For the national level, we conducted interviews in Amman, per telephone and per email with Jordanian government officials, representatives of international donor organisations, academics and staff members of NGOs.

9 Two group discussions were with different refugees from (i) Syria and (ii) Sudan, Somalia, Iraq and Ghana, thus eligible and non-eligible, respectively, for CfW participation. Furthermore, there were two participatory community discussions on the design of a possible, respectively, the completion of a recent CfW project.

10 We analysed data collected by the GIZ project “Improvement of Green Infrastructure” during the first two rounds of their Post-employment Survey, with all 984 and 863 CfW participants in 2019 and 2020, respectively. The survey team included the perspective of non-participants by conducting interviews with participants before they learnt that they would be accepted for participation in the CfW programmes. About a quarter of the respondents were women, almost a half were Syrians.

11 A third of these interviewees at community level were women, a quarter were Syrians (73 per cent Jordanians, 2 per cent other nationalities).

12 For instance, CfW participants renew mutual trust when they repay their debts to local shopkeepers.

13 Only in a second step we asked direct questions on the CfW programmes and their possible effects.

14 Similarly, a survey by NAMA and KAS (2018) found that two-thirds of 600 Syrian respondents felt “to a great extent” welcomed in Jordan and only 1 per cent “to a little extent” or “not at all”. Likewise, two-thirds of the 1,305 Jordanian respondents said they had positive or very positive feelings towards Syrians; only 3 per cent admitted having negative or very negative feelings.

15 These findings are in line with the study by Nama and KAS (2018), in which almost two-thirds of Jordanian respondents, and even a considerable share of Syrians, believed that the presence of Syrian refugees had a negative effect on Jordan’s public services.

16 Roxin et al. (2020) also find that CfW in Turkey affected mainly the sense of belonging of participants.

17 According to social norms, using public transport is considered unsafe and disrespectable for women, thus constituting another barrier to paid employment.

18 Respondents of both nationalities indicated that such reservations are stronger in Syrian society but, as Syrian women added, reservations weakened after their flight to Jordan mainly for economic reasons.

19 For instance, participatory events in Marka and Kafr Asad provided a much valued opportunity to discuss and prioritise different activities to rehabilitate schools, making parents and community members at large more interested in becoming actively involved in future community events and decisions.

20 Individual critics deplored unequal treatment within mixed teams (e.g. if Syrians feel they have to carry out harder tasks than their Jordanian co-workers), damaging trustful relations (expert interview, Amman).

21 Roxin et al.’s panel survey (2020) found that, in Jordan, the trust of both, CfW participants and non-participants, had increased. However, in Turkey, only CfW participants’ trust had slightly increased and non-participants’ trust had decreased, probably due to a more general trend of rising xenophobia in Turkey.
